# Supplementary material for: Polyketide synthase gene clusters in the ericoid mycorrhizal fungus Oidiodendron maius: genome-wide prediction, expression profiling, and functional characterization of knockout mutants
Source: Front Plant Sci. 2026 Sep 16;17:1924430. doi: 10.3389/fpls.2026.1924430 (PMC13623903; doi:10.3389/fpls.2026.1924430)
Supplement: Supplementary file 1 [file SupplementaryFile1.zip › Supplementary Materials/Supplementary Folder 1.zip!/Oidma1_AssemblyScaffolds_Repeatmasked/knownclusterblast/cluster56/jgi.p_Oidma1_46518_mibig_hits.html]

| MIBiG Protein | Description | MIBiG Cluster | MiBiG Product | % ID | % Coverage | BLAST Score | E-value |
| --- | --- | --- | --- | --- | --- | --- | --- |
| ACB12556.1 | Fum10 | BGC0000063 | Fumonisin\_biosynthetic\_gene\_cluster | 25 | 32.6690821256 | 137 | 1.3e-31 |
| ABJ90146.1 | MoeA4 | BGC0000805 | Moenomycin\_biosynthetic\_gene\_cluster | 29 | 24.577294686 | 136 | 3.7e-31 |
| AIL50189.1 | putative\_5-aminolevulinate\_CoA-ligase | BGC0000213 | Colabomycin\_biosynthetic\_gene\_cluster | 27 | 30.0120772947 | 134 | 1.1e-30 |
| AGY30675.1 | Ann3 | BGC0001298 | 4-Z-annimycin\_biosynthetic\_gene\_cluster | 29 | 29.1666666667 | 133 | 2.4e-30 |
| ADC79613.1 | BafX | BGC0000028 | Bafilomycin\_biosynthetic\_gene\_cluster | 30 | 23.0072463768 | 130 | 2e-29 |
| ADI58647.1 | AsuD3 | BGC0000187 | Asukamycin\_biosynthetic\_gene\_cluster | 30 | 22.2222222222 | 125 | 6.5e-28 |
| CAG69514.1 | 2,3-dihydroxybenzoate-AMP\_ligase | BGC0000352 | Fimsbactin\_biosynthetic\_gene\_cluster | 28 | 21.6183574879 | 123 | 3.2e-27 |
| AKD43499.1 | Acyl-CoA\_ligase | BGC0001409 | Dutomycin\_biosynthetic\_gene\_cluster | 30 | 23.9734299517 | 121 | 7.2e-27 |
| ABP55493.1 | thioester\_reductase\_domain | BGC0001006 | Lymphostin\_biosynthetic\_gene\_cluster | 31 | 24.3961352657 | 110 | 1.7e-23 |
| ACN64850.1 | PokL | BGC0001061 | Polyketomycin\_biosynthetic\_gene\_cluster | 31 | 20.229468599 | 108 | 8.2e-23 |
| ACR50792.1 | putative\_acyl-CoA\_synthase | BGC0000163 | Tetronasin\_biosynthetic\_gene\_cluster | 27 | 22.88647343 | 103 | 2.7e-21 |
| CAQ52626.1 | type\_I\_polyketide\_synthase,\_loading\_module\_and\_modules\_1-3 | BGC0001066 | Kendomycin\_biosynthetic\_gene\_cluster | 29 | 21.6787439614 | 101 | 1.3e-20 |
| AAN32979.1 | BarE | BGC0000962 | Barbamide\_biosynthetic\_gene\_cluster | 24 | 23.0676328502 | 100 | 2.2e-20 |
| ACO31297.1 | PtmA3 | BGC0001140 | Platensimycin\_/\_platencin\_biosynthetic\_gene\_cluster | 28 | 23.1280193237 | 100 | 2.2e-20 |
| ADD82992.1 | PtnA3 | BGC0001156 | Platencin\_biosynthetic\_gene\_cluster | 27 | 23.1280193237 | 99 | 3.8e-20 |
| CAI94705.1 | putative\_acid\_AMP\_ligase | BGC0000141 | Rubradirin\_biosynthetic\_gene\_cluster | 28 | 22.4033816425 | 99 | 5e-20 |
| CAE02600.1 | putative\_acyl-coA\_ligase | BGC0000024 | Aureothin\_biosynthetic\_gene\_cluster | 26 | 22.5241545894 | 89 | 3e-17 |
| ADI58645.1 | AsuD1 | BGC0000187 | Asukamycin\_biosynthetic\_gene\_cluster | 24 | 22.1014492754 | 89 | 5.2e-17 |
| AIL50191.1 | putative\_amide\_synthetase | BGC0000213 | Colabomycin\_biosynthetic\_gene\_cluster | 24 | 21.6183574879 | 87 | 1.5e-16 |
| AFK57212.1 | DidA | BGC0000985 | Didemnin\_biosynthetic\_gene\_cluster | 26 | 31.1594202899 | 84 | 9.8e-16 |
| CAO85890.1 | aryl-coA\_ligase | BGC0000110 | Neoaureothin\_biosynthetic\_gene\_cluster | 25 | 21.6787439614 | 84 | 1.7e-15 |
| AFV52184.1 | acyl-CoA\_synthetase/P450\_monooxygenase | BGC0000081 | Kedarcidin\_biosynthetic\_gene\_cluster | 27 | 22.9468599034 | 80 | 1.8e-14 |
| CAI94686.1 | hypothetical\_protein | BGC0000141 | Rubradirin\_biosynthetic\_gene\_cluster | 27 | 24.3961352657 | 78 | 9.1e-14 |
| CBW45647.1 | PI\_synthetase\_3\_and\_4 | BGC0000952 | Pristinamycin\_biosynthetic\_gene\_cluster | 24 | 24.6980676329 | 74 | 1.3e-12 |
| WP\_051700111.1 | non-ribosomal\_peptide\_synthetase | BGC0001368 | Tambromycin\_biosynthetic\_gene\_cluster | 24 | 25.5434782609 | 71 | 1.5e-11 |
| CCJ67639.1 | TaaD | BGC0000447 | Tolaasin\_biosynthetic\_gene\_cluster | 23 | 22.5845410628 | 61 | 1.5e-08 |
| ABR67749.1 | CmnF | BGC0000316 | Capreomycin\_biosynthetic\_gene\_cluster | 24 | 28.5024154589 | 61 | 1.5e-08 |
| CAQ43084.1 | non\_ribosomal\_polypeptide\_synthetase | BGC0000970 | Chondrochloren\_biosynthetic\_gene\_cluster | 22 | 23.9734299517 | 60 | 2e-08 |
| ABB69081.1 | putative\_L-prolyl-AMP\_ligase | BGC0000260 | Prodigiosin\_biosynthetic\_gene\_cluster | 22 | 23.309178744 | 57 | 1.3e-07 |
| ABR67758.1 | CmnO | BGC0000316 | Capreomycin\_biosynthetic\_gene\_cluster | 23 | 20.4106280193 | 51 | 9.2e-06 |
| AAN85530.1 | nonribosomal\_peptide\_synthetase,\_discrete\_adenylation\_domain | BGC0001101 | Leinamycin\_biosynthetic\_gene\_cluster | 25 | 23.6111111111 | 51 | 9.2e-06 |
